# Surface phase transitions and crystal habits of ice in the atmosphere

**DOI:** 10.1126/sciadv.aay9322

**Published:** 2020-05-20

**Authors:** Pablo Llombart, Eva G. Noya, Luis G. MacDowell

**Affiliations:** 1 Instituto de Química Física Rocasolano, Madrid, Spain.; 2 Departamento de Química Física, Universidad Complutense de Madrid, Madrid, Spain.

## Abstract

With climate modeling predicting a raise of at least 2°C by year 2100, the fate of ice has become a serious concern, but we still do not understand how ice grows (or melts). In the atmosphere, crystal growth rates of basal and prism facets exhibit an enigmatic temperature dependence and crossover up to three times in a range between 0° and −40°. Here, we use large-scale computer simulations to characterize the ice surface and identify a sequence of previously unidentified phase transitions on the main facets of ice crystallites. Unexpectedly, we find that as temperature is increased, the crystal surface transforms from a disordered phase with proliferation of steps to a smooth phase with small step density. This causes the anomalous increase of step free energies and provides the long sought explanation for the enigmatic crossover of snow crystal growth rates found in the atmosphere.

## Teaser

Atomic-scale description of the ice structure predicts ice crystal growth rates and explains snow crystal shapes.

## INTRODUCTION

**Fig. 1:**
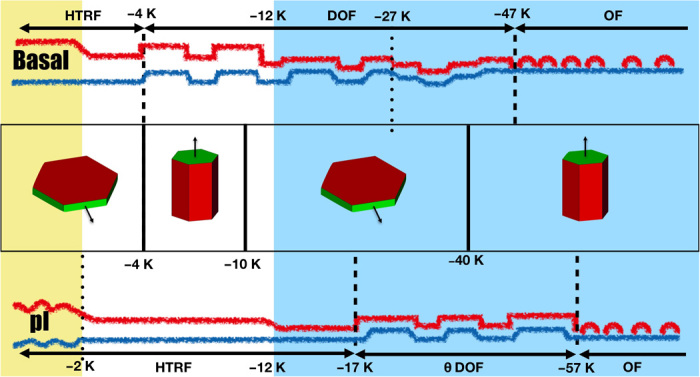
Growth of ice crystals at low supersaturation. (**Middle**) As temperature Δ*T* = *T* − *T_t_* decreases below the triple point, *T_t_*, the habit of hexagonal ice prisms grown in the atmosphere changes sharply from platelike to columnar at ca. −4 K, from columnar to platelike at −10 K, and somewhat less sharply from platelike to columnar at −40 K (*8*). The facet which grows faster as indicated by the arrows, dictates the prevalence of plates or columns. The change of crystal habits results from a crossover in the growth rates of the basal and prism facets. (**Top** and **bottom**) Sketch of surface structural evolution with temperature as found in our work. Blue lines represent the i/f surface, and red lines represent the f/v surface. The basal surface (top row) is a high-temperature reconstructed flat (HT-RF) phase from Δ*T* = 0 to −4 K. It becomes a DOF phase in the range between ca. −4 and −47 K and is transformed into an OF phase at lower temperatures. In this phase, surface disorder resulting from patches of liquid-like molecules remains. The prism surface (bottom row) is an HT-RF phase all the way from 0 to −17 K but is very close to the roughening transition at Δ*T* > − 2 K in our model. In the range from −17 to −57 K, it is a DOF phase and becomes an OF phase below −57 K. At the transition from DOF-like to HT-RF phases, step free energies increase anomalously and result in the crossover of crystal growth rates. The shaded areas illustrate the temperature range where melting of full bilayers has been accomplished. Blue, less than one full bilayer; white, less than two full bilayers; yellow, more than two full bilayers.

The Nakaya diagram documents the hidden mystery of snow crystal growth (*1*): As temperature is cooled down from 0°C to −40°C, ice crystals in the atmosphere change their habit from plates, to columns, to plates and back to columns in a puzzling and unexplained sequence that holds the key to our understanding of the ice surface (Fig. 1).

After heterogeneous nucleation (*2*, *3*), there is still a long way before ice crystallites adopt the micrometer size found in cirrus clouds (*4*). Whether the ice embryos transform into mature columnar or platelike hexagonal prisms depends on the relative growth rate of basal and prism facets (*5*, *6*). But what drives the crossover of basal and prism growth rates, and how such changes are related to the ice surface structure, remains completely unknown to date (*7*, *8*).

Kuroda and Lacmann (*9*) speculated that the occurrence of surface phase transitions could result in sudden changes of the crystal growth rate and provide an explanation for the observed crystal habits, but unfortunately, the experimental verification of this hypothesis has remained elusive to date (*10*–*14*) and our current understanding is still far from providing a molecular explanation (*14*–*17*).

Actually, for a long time, experiments and simulations have been aimed at measuring the onset of premelting and the premelting layer thickness (*10*, *11*, *18*), but the results have been often contradictory and showed a continuous increase of the premelting film, with little signs of a phase transition (*14*). Recent experiments with increased sensitivity show evidence of layering phenomena on the basal facet (*13*), while simulations of the monoatomi Water model (mW) emphasize also the importance of the heterogeneous in-plane structure of ice’s premelting film (*19*). This is a very important observation that highlights the need to probe the structure of ice’s surface not only in the perpendicular direction (layer thickness) but also, in the horizontal direction, because often parallel correlations of the surface structure hold the key to understanding surface properties and crystal growth (*20*–*23*).

One of the main achievements of simple solid on solid (SOS) crystal growth models has been the identification of the roughening transition, which signals the onset of diverging parallel correlations of the surface height fluctuations of a crystal (*20*). Below the roughening temperature, *T*_R_, at an ordered flat (OF) phase, crystal surfaces exhibit only limited disorder, with surface height fluctuations that remain finite and congruent with the underlying bulk lattice (as in the usual Kossel crystal). At *T*_R_, a roughening transition of the Kosterlitz-Thouless type occurs, where the crystal surface unbinds from the underlying crystal mold and exhibits diverging height fluctuations, which do not differ qualitatively from those found at a fluid-fluid interface. Below *T*_R_, at the OF phase, crystal growth is a slow activated process that occurs via two-dimensional (2D) nucleation and is completely suppressed at low saturation. On the contrary, at a rough phase above *T*_R_, crystal growth is no longer activated and depends linearly on saturation (*20*).

This basic framework of crystal growth theory can become far more complicated in extended SOS models, where one incorporates molecular interactions at different length scales (*21*–*23*). Particularly, a preroughening transition at a temperature *T*_pr_ has been documented for some models, where the OF phase “roughens,” meaning that it exhibits up and down correlated crystal step proliferation, but it is “flat” in the sense that the crystal height fluctuations remain finite (*21*). Dynamically, this disordered flat (DOF) phase is thought to exhibit crystal growth behavior intermediate between an OF phase and a rough phase, because growth remains activated in principle, but the step free energies are expected to decrease much as a signature of step proliferation (*21*, *24*). The occurrence of a DOF phase is thought to be accompanied partly with layering (*25*), as observed in experiments on the ice basal facet (*13*), and with strong parallel heterogeneity, as reported for the mW model of water (*19*).

Here, we show that, similar to bulk ice polymorphism, the facets of ice exhibit a number of surface polymorphs as temperature changes. Particularly, we identify a transition from an OF to a DOF phase that is akin to microphase separation of terraces on the crystal surface (*23*, *21*). Upon increasing temperature, the premelting film thickness grows and the surface structure flattens again, resulting in the unexpected increase of nucleation step free energies and the crossover of crystal growth rates exactly as found in laboratory and atmospheric field studies (*8*, *17*).

## RESULTS

**Fig. 2:**
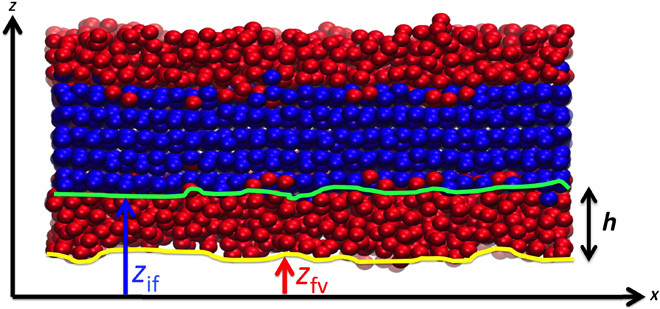
Characterization of the premelting layer. After placing a slab of perfect ice in vacuum, a premelting layer of disordered water molecules is formed spontaneously in a few nanoseconds as shown in the snapshot. Using a suitable order parameter, it is possible to distinguish liquid-like (red) from solid-like (blue) molecules. The state of the premelting film may be described in terms of two different surfaces, *z*_if_(**r**) and *z*_fv_(**r**), separating the film from bulk ice and vapor phases, respectively. A film thickness, *h*(**r**), may be defined as the difference between ice/film and film/vapor surfaces such that *h*(**r**) ***=*** ∣ *z*_fv_(**r**) **−** *z*_if_(**r**)∣.

**Fig. 3:**
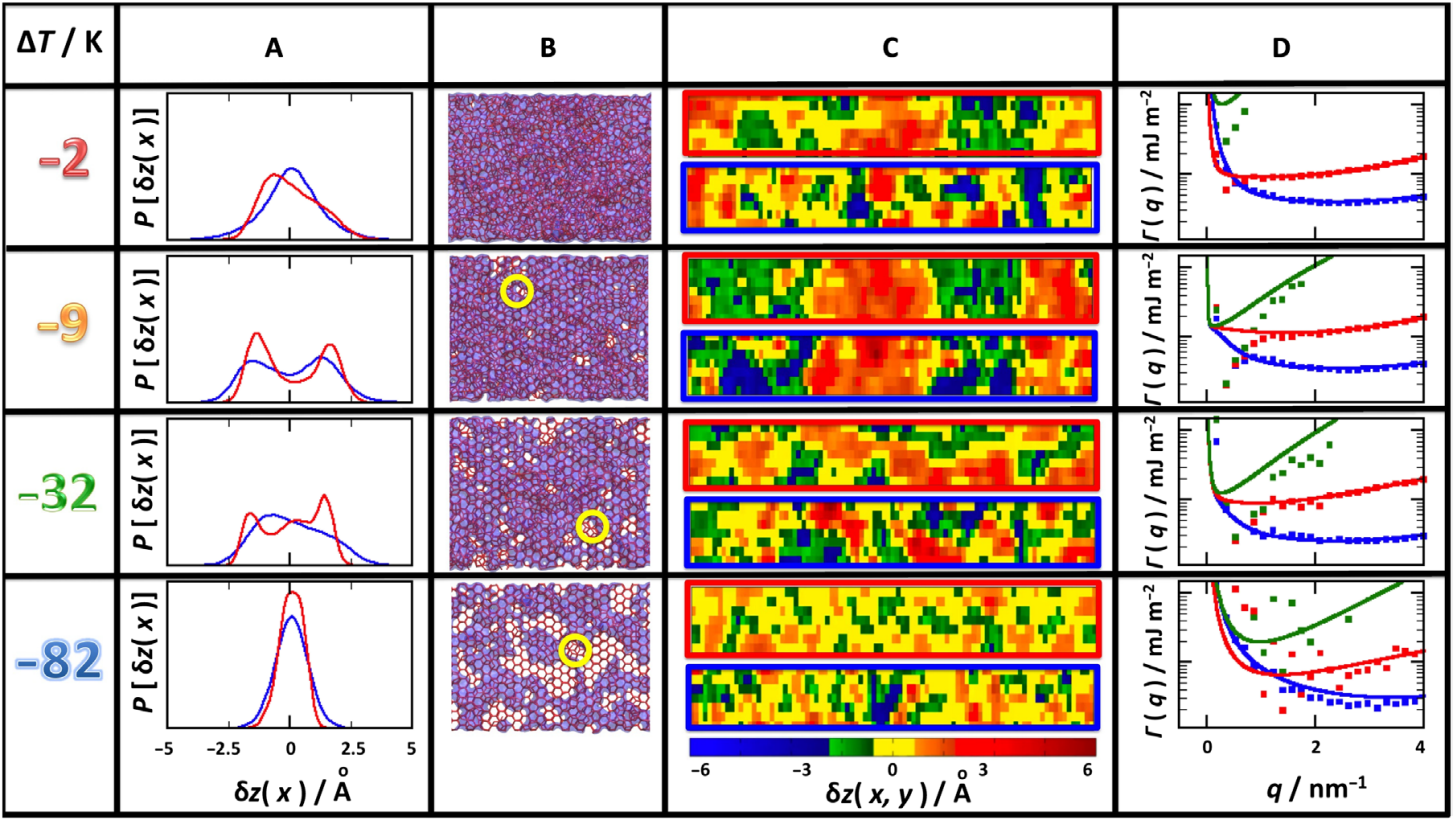
Surface fluctuations on the basal facet. (**A**) Probability distribution of ice/film (blue) and film/vapor (red) surface fluctuations, as measured by the deviations of the interface position *z*_α_(*x*) about the average surface ${\bar{z}}_{\alpha }$, for α = {*if*, *fv*}. Results are shown for different temperatures as indicated in the legend. (**B**) Snapshots of the basal surface at the same four temperatures. Red lines show the connected hydrogen bond network of all solid-like and liquid-like water molecules. The violet patches represent disordered liquid-like molecules. At low temperature, the surface is mainly a regular hexagonal honeycomb with a few patches of liquid-like molecules sitting on interstitial positions (as indicated by the yellow circles). The extent of filled interstitial positions increases as the premelting layer covers the surface. (**C**) Plot showing a snapshot of local surface height fluctuations δ*z*_if_(**r**) (bottom, blue frame) and δ*z*_fv_(**r**) (top, red frame) on the basal ice face. Notice the emergence of large-scale correlated patches for the DOF phase in the temperature range Δ*T* = −32 to −9 K (see movies S1 and S2). The patches disappear at high temperature as the surface flattens again. (**D**) Wave vector–dependent stiffness coefficients, as obtained from the inverse surface structure factor for i/f correlations (blue), f/v correlations (red), and crossed i/f -f/v correlations (green). Crosses are results from simulation; full lines are fits to the SG-CW model. The results show that all surfaces are smooth, as indicated by the divergence of Γ(*q_x_*) as *q_x_* → 0. Note that the sharp minimum appears at intermediate length scales in the temperature range Δ*T* = −32 to −9 K, where the DOF phase is present.

**Fig. 4:**
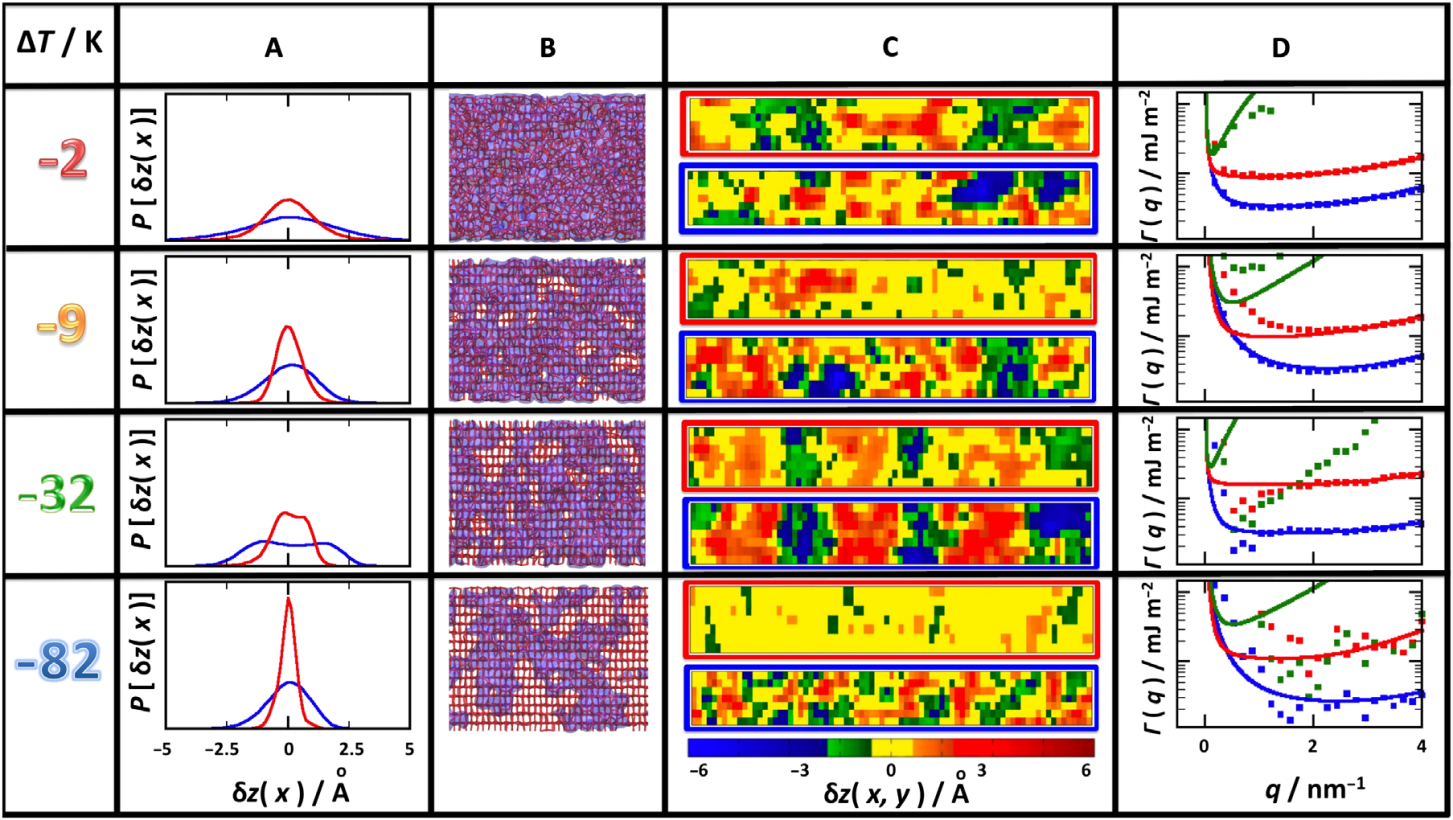
Surface fluctuations on the prism facet. Content displayed as in Fig. 3. (**A**) A bimodal distribution in this facet is only visible at temperature Δ*T* = −32 K. (**B**) Here, the snapshots show the characteristic rectangular mesh of the prism facet. At low temperature, the liquid-like molecules form patches on the solid surface as in the basal face. (**C**) Emergence of large correlated domains signals a DOF phase that is clearly visible at Δ*T* = −32 K and vanishes at notably lower temperatures than for the basal facet (see movies S4 and S5). (**D**) Likewise, the sharp minimum of the stiffness coefficient is visible only at and below Δ*T* = − 32 K.

We perform large-scale computer simulations of the TIP4P/Ice point charge model of water (*26*) on elongated rectangular surfaces meant to characterize large wavelength correlations along one direction. A bulk ice sample is placed in vacuum at temperatures below the model’s triple point, *T*_t_ = 272 K. After a few nanoseconds, the surface spontaneously develops a layer of quasi-liquid disordered molecules that can be readily distinguished from the underlying bulk crystal network (Fig. 2). Averaging the position of the outermost solid and liquid-like atoms of the premelting layer about points **r** on the plane of the interface, we are able to identify distinct ice/film (i/f) *z*_if_(**r**) and film/vapor (f/v) *z*_fv_(**r**) surfaces (*27*), which separate the premelting film from the bulk solid and vapor, respectively (Fig. 2 and Materials and Methods). Using these definitions, we can monitor the local thickness of the premelting film as *h*(**r**) = ∣ *z*_fv_(**r**) − *z*_if_(**r**)∣ and perform a detailed study of in-plane surface structure as a function of undercooling, Δ*T* = *T* − *T*_t_. The thermally averaged thickness of the premelting layer, *h*, grows from about 3 Å at Δ*T* = − 82 K to 9 Å at Δ*T* = − 2 K with little measurable anisotropy (fig. S1) (*18*). However, as recently observed for the basal plane in the mW model (*19*), this thin disordered layer is laterally inhomogeneous up to about Δ*T* = − 9 K (Fig. 3B and fig. S2). Our study reveals that the heterogeneity is also found to a similar extent on the prism plane, with little qualitative differences (Fig. 4B and fig. S3).

Where does the marked crystal rate anisotropy found in experiments come from? To shed light into this problem, we quantify surface fluctuations along the long direction, *x*, of the simulation box. This is done by monitoring deviations, δ*z*(*x*), of the local surface position, *z*(*x*), from the instantaneous average surface, $\bar{z}$, for either the ice/film $\delta z_{\text{if}}(x)=z_{\text{if}}(x)-{\bar{z}}_{\text{if}}$ or the film/vapor surface $\delta z_{\text{fv}}(x)=z_{\text{fv}}(x)-{\bar{z}}_{\text{fv}}$ (Materials and Methods). This analysis is essential to reveal differences between basal and prism planes and allows us to identify a number of phase transitions along the sublimation line, which cannot possibly be inferred from visual inspection of the snapshots (Figs. 3B and 4B and figs. S2 and S3) or the premelting layer thickness (fig. S1).

### Structure of the basal facet

At low temperature (Δ*T* = −82 K), the basal plane consists of a relatively OF solid surface, as revealed by a singly peaked, close to Gaussian distribution of both i/f and f/v surface fluctuations (Fig. 3A and fig. S4A). From the snapshots (Fig. 3B), the solid surface is formed mainly of an oxygen-unreconstructed stack of chair hexagons (*28*–*30*). Patches of disordered liquid-like molecules are found and often show a tendency to sit on interstitial positions at the center of the primary hexagonal mesh (as in the so-called Honeycomb Fletcher phase) (*28*). The distributions remain unimodal up to Δ*T* = −52 K, but somewhat broaden, revealing a large increase of disorder in this temperature interval, which is consistent with observations of sum frequency generation spectroscopy (*10*). At a temperature Δ*T* = −42 K close to the onset of substantial molecular mobility of the outermost surface layer (*31*), the distribution of δ*z*_fv_(*x*) develops a distinct trimodal character (fig. S4A). A main peak is centered at the mean surface position, and two other peaks appear to the left and right. The central peak of the trimodal distribution for δ*z*_fv_(*x*) gradually fades away into a bimodal, which persists up to Δ*T* = −6 K. In a narrow range between −9 and −6 K, the distributions of δ*z*_fv_(*x*) and δ*z*_if_(*x*) are both fully bimodal and congruent. Last, at the temperature of −2 K, the bimodal collapses sharply into one single unimodal distribution.

From our analysis, the outer f/v surface of the premelting film exhibits a bimodal distribution centered at the mean surface location all the way from −22 to −6 K. The onset of bimodality very much correlates with the vanishing of the (ppp-polarized) dangling OH bond stretch observed in sum-frequency generation (SFG) spectroscopy experiments (*10*). The separation between peaks in the bimodal is approximately 3.1 Å, somewhat smaller than *c*/2 = 3.65 Å, the distance between adjacent bilayers.

Furthermore, the bimodal evolves after the appearance at low temperature of a trimodal distribution with a main peak centered at the mean surface position, as if ice melting resulted in the formation of water-like molecules at half-integer lattice positions (fig. S4A).

### Preroughening and smoothening transitions

A strongly disordered phase consisting of a smooth surface with large-scale step proliferation has been documented in the literature for SOS models and is known as a DOF phase (*21*). Exactly as suggested by the extended SOS model, we observe that the low-temperature smooth flat phase with unimodal probability distribution transforms into a locally rough interface with a trimodal probability distribution. This change is the hallmark of a preroughening transition (*21*, *25*). Here, the low-temperature flat phase (OF) is transformed into a DOF phase at a preroughening temperature Δ*T*_pr_ ≈ −47 K. However, contrary to the usual scenario, at high temperature, the DOF phase does not undergo roughening but rather transforms across an apparently first-order surface phase transition into a new flat phase with unimodal distribution at a smoothening temperature, Δ*T*_s_ ≈ −4 K (Fig. 3A). This transition has not been previously reported but is consistent with existing surface phase diagrams for the extended SOS model (*22*, *25*). Following the notation of SOS literature, we call this a high-temperature reconstructed flat (HT-RF) phase. In practice, the nature of the actual HT-RF phase found here for the basal facet can be inferred from the snapshots (Fig. 3B and fig. S2). A premelting film appears to be fully developed, and the underlying hexagonal mesh of solid molecules is covered with interstitial water molecules located at the center of the hexagons.

**Fig. 5:**
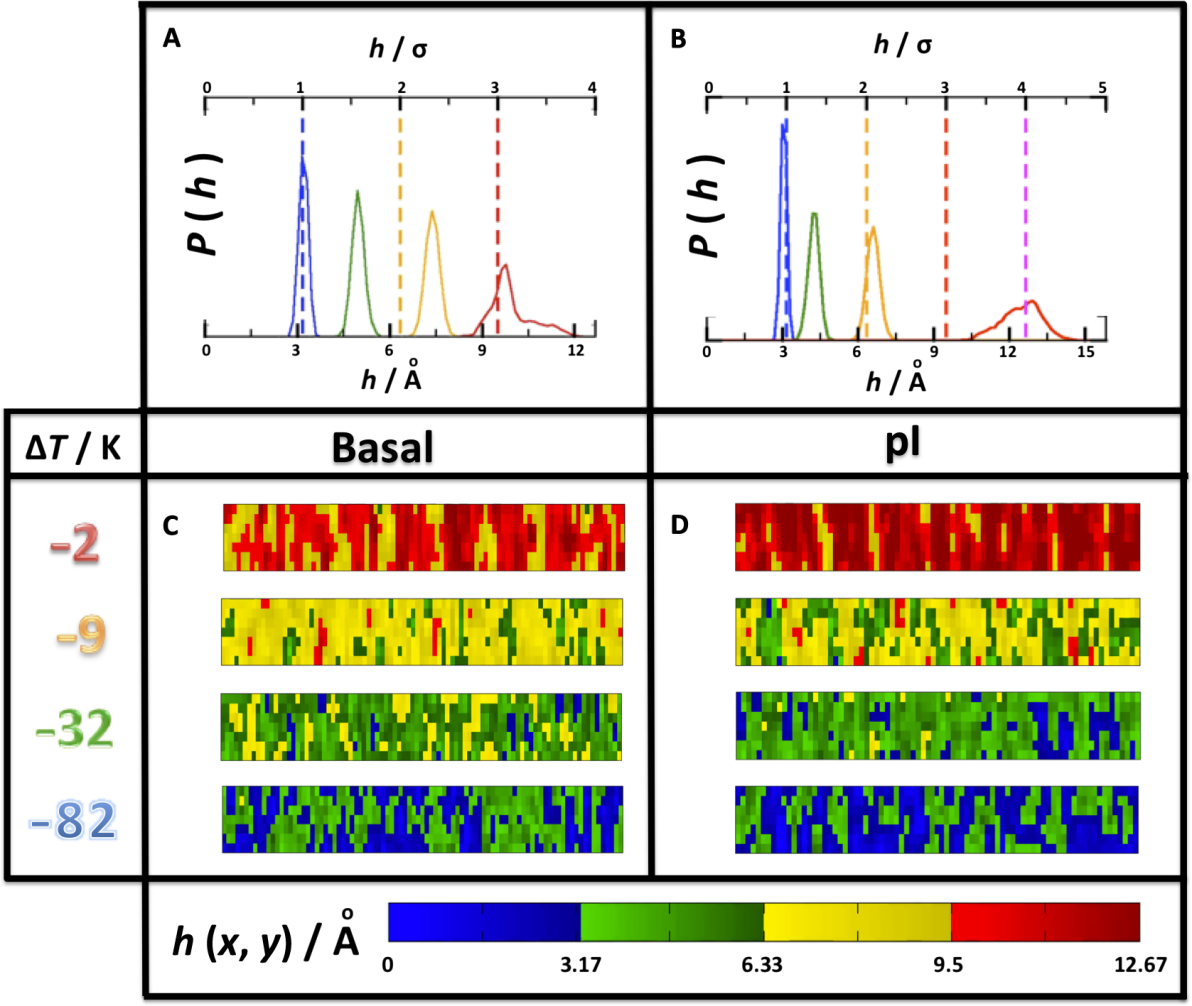
Fluctuations of premelting thickness. Results are shown for basal (**A** and **C**) and prism (**B** and **D**) facets. (A and B) Distribution of average film thickness, *h*, as a function of undercooling, with Δ*T* = −82 K (blue), −32 K (green), −9 K (yellow), and −2 K (red). Dashed vertical lines represent full layers in units of the molecular diameter. The transition of premelting layer thickness across integer multiples of the molecular diameter occurs at broadly the same temperature for the basal and prism facets. (C and D) Surface plot of the instantaneous premelting thickness, *h*(**r**), for basal (C) and prism (D) surfaces (see movies S3 and S6). Notice the absence of large correlated domains at all temperatures, in marked contrast with the δ*z*_if_(**r**) and δ*z*_fv_(**r**) surfaces shown in Figs. 3 and 4.

As expected for the preroughening scenario predicted in extended SOS models (*23*, *25*), the transition to a DOF phase is strongly correlated with the growth of a premelting film in a loosely layer-wise fashion. This can be shown by inspecting the distribution of laterally averaged film thickness, *P*(*h*), in Fig. 5A (see also fig. S4B). We find that the stabilization of the bimodal DOF phase observed at about Δ*T* = −22 K results after the full formation of a second layer of premelted ice, as revealed by a shift in the maximum of the distribution from *h* = 5.0 Å at Δ*T* = −32 K to *h* = 7.4 Å at Δ*T* = −9 K. SFG experiments reveal a large increase in the premelting film thickness in this temperature range (*13*). Likewise, the transition from a DOF phase into the HT-RF phase is also accompanied by the formation of a third premelted layer, as revealed by the shift in the premelting layer thickness from *h* = 7.4 Å at Δ*T* = −9 to *h* = 9.8 Å at −2 K (Fig. 5A).

The DOF phase is not only characterized by step proliferation and strong local disorder. Predictions from the extended SOS model also show that the steps are highly correlated, while, depending on the system, the preroughening transitions can be either second-order (accompanied by diverging parallel correlations) or first-order (with parallel correlation that remain finite) (*25*). We explore the extent of parallel correlations with a plot of the local surface height fluctuations of the ice/film δ*z*_if_(**r**) and film/vapor δ*z*_fv_(**r**) surfaces (Fig. 3C and fig. S6, A and B). At low temperature, the i/f surface exhibits small amplitude up and down domains, with small correlation lengths. At Δ*T* = −9 K, where the DOF phase is fully formed, we observe very large up and down domains of about 9 nm in length that remain correlated over the full simulation box, as is visible both in the figure and in the accompanying movies (movies S1 and movies S2). However, at Δ*T* = −2 K, just a few kelvin above, the large correlated domains vanish. Our results are consistent with glancing angle x-ray experiments, which reported the appearance of a large surface correlation length in the nanometer range, and the sharp disappearance of the long-range correlations close to the triple point (*32*).

The nature of these correlations can be quantified from the wave vector–dependent surface structure factor. Plots of the related effective stiffness $\Gamma_{\alpha \beta }(q_{x})=\frac{k_{B}T}{A\langle z_{\alpha }(q_{x})z_{\beta }(q_{x})\rangle q_{x}^{2}}$ are shown in Fig. 3D (fig. S4C). The results confirm that both at the preroughening and at the smoothening transition, the parallel correlations remain finite at *q_x_* → 0, as can be inferred from the strong divergence of the effective stiffness coefficients (notice the logarithmic scale of the figure). Everywhere in the region where a DOF phase is present, however, the strongly correlated up and down domains are detectable as a sharp and deep minimum of the stiffness coefficients at intermediate wave vectors (i.e., as peaks in the surface structure factor). A full theoretical description of this strong enhancement of large but finite correlations seems difficult. However, we can definitively observe in our results how the location of the sharp minima at intermediate wave vectors decreases as the size of the correlated domains in Fig. 3C increases.

### Structure of the prism facet

The study of surface fluctuations on the prism facet is substantially different. A low-temperature flat phase that preserves the expected low-temperature rectangular mesh is observed at Δ*T* = −82 K (Fig. 4B). At this temperature, the distribution of both *z*_if_(*x*) and *z*_fv_(*x*) is unimodal but becomes gradually broader and skewed to the left as temperature is increased (fig. S5A). At Δ*T* = −32 K, however, the distribution of *z*_if_(*x*) and *z*_fv_(*x*) becomes slightly bimodal. From Δ*T* = −12 K to Δ*T* = −2 K, the distribution becomes again completely unimodal and Gaussian like, but has broadened abruptly at Δ*T* = −2 K, signaling the approach of a roughening transition (*20*). Although the order parameter is not sharp enough to reveal a strong bimodality, a large number of steps are formed in the range between −62 and −22 K with distributions that seem to resemble a θDOF phase [similar to the DOF phase, but with a continuous change of the step coverage (*25*)]. The transition from a DOF phase to a new HT-RF phase is visible in the surface maps (Fig. 4C), where large-scale domains appear to end at −32 K. This is also visible in the surface structure factors depicted in Fig. 4D, which reveal again diverging stiffness coefficients at *q_x_* → 0 (whence, flat phase), and the complete disappearance of the sharp minimum beyond this temperature (whence, loss of long-range surface order). The transformation of the surface structure across the θDOF phase is also accompanied with continuous increase of the premelting film (Fig. 5B), but the transition from two to three layers and beyond appears to occur almost at the same temperature as in the basal facet. On the contrary, the transition from the θDOF phase to the HT-RF phases is distinctly different at the basal (ca. *T*_s_ = −4 K) and prism (ca. *T*_s_ = −27 K) facets.

We find that, on average, for both the basal and prism facets, *z*_fv_(**r**) follows broadly the corrugation imposed by the *z*_if_(**r**), and the fluctuations of premelted film thickness appear as broad unimodal distributions with no sign of bimodality or diverging correlation lengths (Fig. 5, A and B). Accordingly, there are apparently no layering transitions along the sublimation line in the temperature range studied, at least in the thermodynamic sense. The lack of large correlation lengths or bimodality is very clearly observed in the surface plots of local film thickness *h*(**r**) = *z*_fv_(**r**) − *z*_if_(**r**) (Fig. 5, C and D; see also figs. S6C and S7C and movies S3 and S6) and is consistent with SFG experiments and preliminary indications for the mW model (*12*, *19*).

### Step free energies

The importance of DOF phases has been well documented (*21*–*23*). The step proliferation is akin to a strong reduction of the step free energy and the sharp decrease of the threshold for linear growth (*21*, *22*, *24*). It is therefore expected that, as temperature rises across the smoothening transition from a DOF to HT-RF phase, the crystal growth rates will decrease anomalously.

**Fig. 6:**
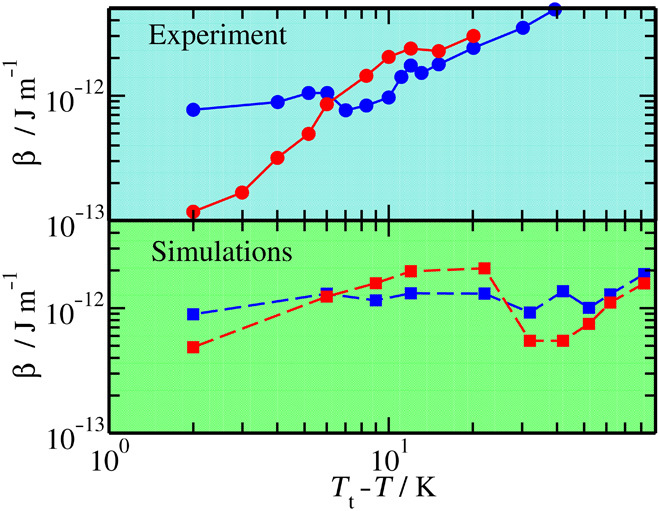
Calculated step free energies. Step free energies as obtained from a fit of the mean field SG-CW model to the regular (Gaussian) part of the stiffness coefficients in Figs. 3D and 4D for the basal (blue) and prism (red) facets. Results (squares with dashed lines) are compared to experimental data (circles with full lines) (*17*) and displayed on two different figures to avoid crowding. Notice that the scale in both figures is the same. The step free energies from the fit exhibit a crossover at ca. −6 K, 2 K above that found in the accepted Nakaya diagram, and display overall a trend similar to the experimental data.

We provide a quantitative test of this expectation using a model of coupled capillary wave and sine-Gordon Hamiltonians (SG-CW) for the spectrum of surface fluctuations discussed recently (*27*). The capillary wave Hamiltonian describes the f/v surface fluctuations, whereas the likelihood of step proliferation in the underlying i/f surface is described with a sine-Gordon Hamiltonian. Both models are coupled with an interface potential that sets the equilibrium film thickness as the difference between *z*_fv_(**r**) and *z*_if_(**r**). The model can be fit to the regular (Gaussian) part of the surface stiffness (Figs. 3D and 4D) and provides phenomenological coefficients for the surface tension and the step free energy, β (Materials and Methods). The nonmonotonous behavior observed for β correlates with the behavior measured experimentally for terrace spreading rates (*5*) and is qualitatively similar to step free energies obtained from growth measurements on snow crystals (Fig. 6) (*17*). For the prism facet, β increases sharply in the range Δ*T* = 20 to 30 K, somewhat below the estimated smoothening temperature. For the basal facet, on the other hand, β increases in the range between 9 and 6 K, very close to the estimated smoothening transition. Although the increase for the basal face is small, it is sufficient to trigger the crossover of step free energies at about Δ*T* = −6 K, in almost exact agreement with the crossover observed in the experimental results (Fig. 6). Accordingly, our results lend support to the suggested scenario of anomalous increase of β in the neighborhood of the smoothening transitions of basal and prism facets, respectively.

## DISCUSSION

### Origin of the anomalous behavior

The amazing polymorphism of ice crystals results from several factors that are unique to water. (i) A subtle balance between proton ordering, which is usually favored enthalpically, and proton disordering, which is favored entropically; (ii) a large number of oxygen lattices consistent with hydrogen bonding; and (iii) the allowance of interpenetrated hydrogen bond networks. A combination of these factors appears to operate also at the ice surface, which, contrary to bulk solid phases, can also gain additional entropy by disruption of the ordered oxygen network.

Below 200 K, quantum mechanical calculations indicate that both basal and prism facets of pristine ice form stripes of alternating up and down dangling hydrogen bonds in a low-temperature highly ordered Fletcher phase (*14*, *29*, *30*). As temperature increases, entropy can be gained by disordering the hydrogen bond network while keeping oxygen positions fixed or, alternatively, by disrupting the oxygen framework altogether to gain translational entropy. The structural evolution of the ice surface as temperature increases results from an intricate interplay of these two factors, which appear to participate successively.

As the OF phase is heated, the surface free energy can be minimized by disordering the dangling hydrogen bond network, at the cost of breaking the stabilizing Fletcher stripes. This loosens the ice surface, resulting in the decrease of step free energies observed at low temperature (see Fig. 6). Once the surface hydrogen bond network has achieved full disorder, however, no further entropy gain can be achieved without much disruption of the oxygen lattice. This provokes the gradual melting of a bilayer and increases the premelting layer thickness (*13*, *18*, *31*). For thin films, this occurs with minimal enthalpy cost because the premelted water molecules can shift to interstitial sites corresponding to an interpenetrated crystal network (see Fig. 3C).

As large amounts of translational entropy are gained by the formation of a thin premelting layer, the surface enthalpy can be lowered by partially building up the hydrogen bond order again. This results in an increase of the local surface stiffness and the rise of the step free energy at ca. Δ*T* = −30 K for the basal face and ca. Δ*T* = −20 K for the prism face observed in Fig. 6.

With hydrogen bond order building up, it is expected that local domains of enhanced surface polarity can result (*14*). These domains are stabilized locally because of the hydrogen bond connectivity of the underlying bulk crystal network. However, at large distance, domains of equal polarity repel each other. The combination of short-range attractive and long-range repulsive interactions is precisely the main ingredient in the stabilization of a DOF phase (*21*, *22*). The entropy cost of local ordering the hydrogen bond network is balanced by the presence of substantial translational disorder of the premelting film and the meandering entropy associated with the moving boundaries of the domains. The gain in entropy is sufficient to drive the line tension between domains effectively negative and promote the spontaneous formation of steps on the surface (*19*).

Similarly, in colloidal science, colloids with short-range attractive and long-range repulsive forces are known to microphase separate. Therefore, the transformation of the low-temperature stripe phase into a DOF phase with compact clusters of different height can be viewed as akin to the transition from lamellar to cluster phases in colloids with competing interactions.

However, the topology of the basal and prism surfaces dictates very different enthalpy costs for the boundary between different domains. For the stripe phase of the basal facet, grain boundaries between locally ordered domains have a very low energy penalty (*28*). For the prism face, on the other hand, the cost of surface grain boundaries between ordered domains is very high (*28*) and does not allow for the stabilization of the DOF phase in such a large temperature range.

Eventually, as the triple point is approached, a new bilayer melts and the meandering entropy of the basal face is traded for the translational entropy gained by the additional layer in the premelted film. In this situation, the ice/film surface is similar to the ice/water interface, and step free energies approach the value expected for ice freezing from water at the triple point (*9*). There, the basal face is faceted, and has a finite step free energy, but the prism face is rough and has a vanishing step free energy (*27*), leading to the observed crossover of step free energies at high temperature (see Fig. 6).

### Relation to crystal growth

Ice crystallites in the atmosphere are expected to grow by a 2D nucleation process, with crystal growth rate, *j*, given by (*9*)

$$j=A(T)S^{m}\text{exp}\;(-g_{m}\frac{\beta^{2}a}{k_{B}^{2}T^{2}S})$$ (1)

where *A*(*T*) is a temperature-dependent prefactor, *S* = ln *p*/*p*_c_ is the saturation, *a* is the surface area per molecule, *g_m_* is a numerical factor, *k*_B_ is Boltzmann’s constant, *p* is the ambient water vapor pressure, and *p*_c_ is the ice/vapor saturation pressure.

For a mononuclear mechanism, the exponent *m* is unity, and *g_m_* = π, whereas for polynuclear growth, *m* = 2/3 and *g_m_* = π/3. In the nucleated regime at constant ambient conditions, the crystal growth rate is therefore dictated essentially by β. If the step free energy of the basal facet is larger than that of the prism facet, the basal face grows slower than the prism face and platelike crystals form. On the contrary, if the basal step free energy is smaller, the basal face grows faster and columnar crystals form (*7*, *9*, *17*).

Close to the melting point, our results show that the prism facet has smaller step free energies than the basal facet (Fig. 6). In this range, accordingly, growth of plates rather than columns is predicted to occur, in agreement with experiments (see Fig. 1). As the temperature decreases, our calculated step free energies cross over twice, predicting correctly the sequence of transitions from plates to columns and back to columns that is the most striking feature in the Nakaya diagram. The first crossover, corresponding to the transition from plates to columns, takes place at Δ*T* = −6 K, which is 2 K away from the value of Δ*T* = −4 K observed in experiments. The next crossover is predicted to occur at the temperature Δ*T* = −26 K, which is shifted by about 15 K from the value of Δ*T* = −10 K in the Nakaya diagram.

This suggests that the precise location of the remaining crystal habit transitions could depend not only on step free energies but also on additional factors, such as the surface mobility of premelted molecules that is embodied in the prefactor, *A*(*T*), or the diffusion limited vapor field that surrounds the crystal (*9*).

In conclusion, we show that in the range of about 80 K below the melting point, the main facets of ice may be found in up to three different surface phases with varying degree of surface disorder, as postulated by Kuroda and Lacmann with amazing intuition almost 40 years ago (*9*). The accompanying phase transitions provide the mechanism for a nonmonotonous change of the relative step free energies for 2D nucleation. Most notably, we observe a premelting mediated process of surface smoothening, which results in the anomalous increase of step free energies. This results in the crossover of relative growth rates of basal and prism facets that is required to explain the Nakaya diagram.

The explanation of the long-standing problem of snow crystal shapes (Fig. 1) proofs that we have now a close to complete molecular description of the surface structure and crystal growth rates of ice in the atmosphere, with immediate implications in atmospheric sciences, glaciology, and climate modeling.

## MATERIALS AND METHODS

### Force field

Our study is performed with the TIP4P/Ice model of water (*26*). This model was purposely designed to best describe the properties of ice. It predicts a melting point of *T* = 272 K, in excellent agreement with experiment, and reproduces the most relevant surface properties at this temperature, such as liquid-vapor surface tension (γ*_lv_* = 82 mN/m calculated by ourselves, compared with γ*_lv_* = 75.7 mN/m from experiment) and solid-liquid surface tension [γ*_sl_* = 29.8 mN/m from (*33*) compared with recommended results γ*_sl_* = 28 mN/m by Pruppacher and Klett (*34*)].

The precise location of the surface phase transitions observed here could somewhat change with the molecular model used, but we expect the generic features to be quite generally observed for other accurate intermolecular potentials.

### Initial configurations

Initial configurations are prepared from a perfect unit cell in pseudo-orthorhombic arrangement, consisting of two layers of hexagonal rings perpendicular to the hexagonal *c* axis and a total of 16 water molecules. For the basal interface, we arrange a stack of 46 × 8 × 8 cells of 47,104 molecules, with the long direction, *x*, aligned along the *b* axis of the pseudo-orthorhombic cell, corresponding to the so-called (basal)[pII] surface arrangement described by Davidchack *et al.* and used in our previous work (*27*, *35*, *36*).

For the prism facet (pI), the simulation box is prepared from a stack of 40 × 8 × 8 unit cells of *N* = 40,960 molecules, with the long direction, *x*, aligned along the *a* axis. This corresponds to the (pI)[basal] arrangement in our recent work. For each such arrangement, we prepare an independent hydrogen bond network as described in (*37*). Using this method, a hydrogen bond network satisfying the Bernal-Fowler rules is selected randomly on a subsystem. This arrangement is replicated four times to obtain the full system. Initial configurations are selected such that the net dipole moment is smaller than 0.4% of one single TIP4P/Ice molecular dipole moment. Notice that in a bulk simulation of ice, hydrogen bond networks are arrested and no further sampling is possible. However, for simulations where the bulk ice phase terminates at a free boundary, as here, ring rotations take place spontaneously during the molecular dynamics simulations and the sampling is performed over many realizations of the hydrogen bond disorder.

After forming the ice slab, we perform NpT simulations of the bulk solid at the desired temperature to obtain equilibrated unit cell dimensions. The solid is then scaled to the average equilibrium cell value, placed in vacuum, and equilibrated again in the canonical ensemble under periodic boundary conditions.

### Computation details

Large-scale simulations are carried out on the MareNostrum 4 facility at Barcelona Supercomputing Center from the Spanish National Supercomputing Network. Classical molecular dynamics simulations are performed with the 2016.4 version of the GROMACS package. Trajectories are evolved with the GROMACS default leap-frog algorithm. Both the Lennard-Jones and Coulomb interactions are truncated at 0.9 nm. The electrostatic interactions are calculated using the particle mesh Ewald method. Simulations are thermostated with the velocity rescale algorithm (*38*) and the Berendsen barostat when required. A relaxation time of 2 ps is used for both the thermostat and barostat. The time step used is 3 fs. This is a somewhat larger time step than 2 fs usually used in thermostated dynamics of TIP4P models, but we have checked that energy is well conserved for time lengths sufficiently larger than the thermostat relaxation time of 2 ps. We believe that the need to perform reliable ensemble averages with correlation lengths in the scale of several many nanometers warrants an increase in the time step. For symplectic algorithms, it is known that this effectively provides the correct dynamics for an effective Hamiltonian with corrections of order *dt*^2^. For this reason, the transitions that we find could be shifted by 1 or 2 K from those obtained for the exact Hamiltonian. Three runs (300 ns long) are launched for each temperature and crystal facet starting from an independent hydrogen bond network. We discard the first 75 ns of each run and perform averages over the remaining 225 ns. Accordingly, our results report thermal averages collected over a total of 675 ns for each temperature and facet.

### Surface analysis

#### Order parameter

Before determining the i/f and f/v surfaces, we label water molecules as either solid or liquid like, using the ${\bar{q}}_{6}$ parameter (*39*). Water-like molecules are those with a ${\bar{q}}_{6}$ parameter below a threshold ${\bar{q}}_{6}^{*}(T)$. To determine the threshold, we simulate the probability distribution of ${\bar{q}}_{6}$ at a number of temperatures in either bulk solid or liquid water. The threshold value ${\bar{q}}_{6}^{*}(T)$ is determined such that the number of mislabeled liquid molecules on the solid phase is equal to the number of mislabeled solid molecules on the liquid phase. Previously, we have shown that using these criteria, we are allocating ice allotropes (ice clathrate and ice Ic environments) as well as surface undercoordinated molecules (mainly interfacial ice Ih) that appear at the ice surface as belonging to the premelting layer. Whence, our ice-liquid surface separates the strict hexagonal ice template from other less ordered interfacial environments. We have found that the number of molecules allocated to ice clathrate, ice Ic, and interfacial ice Ih environments does not appear to change much in the temperature interval studied here so that the premelting layer fluctuations are hardly affected by this choice (*40*).

#### Surface location

A cluster analysis is performed to determine which molecules pertain to the condensed phase. Water molecules with oxygen atoms at a distance less than 3.5 Å belong to the same cluster. The i/f surface is determined from the positions of solid-like atoms in the largest solid cluster. We use the heights of the four topmost (or bottommost) solid atoms of the upper (lower) interface. At a given point **r** on the plane of the interface, we find all the solid-like atoms lying within a rectangular prism centered at **r**. The base of the prism is half the area of the pseudo-orthorhombic unit cell. The surface height *z*_if_(**r**) at that point is determined from the average location of the four uppermost solid-like atoms. At the same point, the liquid surface for the upper (lower) interface is determined by averaging the position of the uppermost (bottommost) four liquid-like molecules of the cluster of condensed molecules lying within a rectangular area of 3σ × 3σ Lennard-Jones molecular diameters. The surfaces *z*_if_(**r**) and *z*_fv_(**r**) are determined over points on a grid on the plane of the interface. The grid has twice as many points as unit cells along the *x* direction and just as many points as unit cells along the *y* direction. We perform the surface analysis from the set {*z*_if_(**r**)} and {*z*_fv_(**r**)} of points on the grid (*27*). The instantaneous mean position of the i/f surface ${\bar{z}}_{\text{if}}$ is determined as the lateral average of {*z*_if_(**r**)} overall points on the grid. From this value, we obtain $\delta z_{\text{if}}(r)=z_{\text{if}}(r)-{\bar{z}}_{\text{if}}$. The laterally averaged fluctuations δ*z*_if_(*x*) are obtained from δ*z*_if_(**r**) upon averaging along points *y*. δ*z*_fv_(**r**) and δ*z*_fv_(*x*) are obtained likewise. Fourier transforms of δ*z*_if_(*x*) and δ*z*_fv_(*x*) are obtained by summing δ*z*_α_(*x*)*e*^−*iqxx*^ over all points along *x*. We have shown previously that the fluctuations of i/f and f/v surfaces so defined consistently provide the expected ice/water and water/vapor surface tensions as the premelting layer thickness grows on approaching the triple point (*27*).

Instantaneous local film heights are obtained as *h*(**r**) = *z*_if_(**r**) − *z*_fv_(**r**). The instantaneous average film thickness is obtained from the mean of *h*(**r**) over the points of the grid.

### SG-CW model and fit

We describe the coupled i/f and f/v surface fluctuations with an extended sine-Gordon model for the i/f surface and the capillary wave model for the f/v surface (*20*). The two terms are coupled via the interface potential, *g*(*h*), with the premelting film thickness given by *h*(**r**) = *z*_fv_(**r**) − *z*_if_(**r**). The full Hamiltonian is given by

$$\begin{array}{c} H=\int dr[\frac{{\tilde{\gamma }}_{\mathrm{iw}}}{2}{\left(\nabla z_{\text{if}}\right)}^{2}+\frac{\gamma_{\mathrm{wv}}}{2}{\left(\nabla z_{\text{fv}}\right)}^{2}+\\ \gamma_{\mathrm{iv}}\nabla z_{\text{if}}\cdot \nabla z_{\text{fv}}-u\text{cos}\;(q_{z}z_{\text{if}})+g(z_{\text{fv}}-z_{\text{if}})] \end{array}$$ (2)

where ${\tilde{\gamma }}_{\mathrm{iw}}$ is the bare stiffness coefficient, γ*_wv_* is the water-vapor surface tension, γ*_iv_* dictates the coupling of surface deformations, *u* accounts for the cost of moving the surface *z*_if_ away from integer lattice spacing, *g*(*h*) is the interface potential dictating the equilibrium film thickness, and *q_z_* is the wave vector for a wavelength equal to the lattice spacing. This Hamiltonian can be expanded to quadratic order in deviations away from the mean surface positions ${\bar{z}}_{\text{if}}$ and ${\bar{z}}_{\text{fv}}$ and yields for the thermally averaged surface fluctuations (*27*)

$$\begin{array}{cc} \langle |z_{\text{if}}^{2}(q)|\rangle & =\frac{k_{B}T}{A}\frac{g^{\prime \prime }+\gamma_{\mathrm{wv}}q^{2}}{[\upsilon +g^{\prime \prime }+{\tilde{\gamma }}_{\mathrm{iw}}q^{2}][g^{\prime \prime }+\gamma_{\mathrm{wv}}q^{2}]-{\left[g^{\prime \prime }+\Delta g^{\prime \prime }-\gamma_{\mathrm{iv}}q^{2}\right]}^{2}}\\ \langle |z_{\text{fv}}^{2}(q)|\rangle & =\frac{k_{B}T}{A}\frac{\upsilon +g^{\prime \prime }+{\tilde{\gamma }}_{\mathrm{iw}}q^{2}}{[\upsilon +g^{\prime \prime }+{\tilde{\gamma }}_{\mathrm{iw}}q^{2}][g^{\prime \prime }+\gamma_{\mathrm{wv}}q^{2}]-{\left[g^{\prime \prime }+\Delta g^{\prime \prime }-\gamma_{\mathrm{iv}}q^{2}\right]}^{2}}\\ \langle z_{\text{if}}(q)z_{\text{fv}}^{*}(q)\rangle & =\frac{k_{B}T}{A}\frac{g^{\prime \prime }+\Delta g^{\prime \prime }-\gamma_{\mathrm{iv}}q^{2}}{[\upsilon +g^{\prime \prime }+{\tilde{\gamma }}_{\mathrm{iw}}q^{2}][g^{\prime \prime }+\gamma_{\mathrm{wv}}q^{2}]-{\left[g^{\prime \prime }+\Delta g^{\prime \prime }-\gamma_{\mathrm{iv}}q^{2}\right]}^{2}} \end{array}$$ (3)

where *g*′′ is the second derivative of the interface potential at the equilibrium film thickness, Δ*g*′′ accounts for enhanced coupled compression-expansion of the film thickness, and $\upsilon =q_{z}^{2}u$. In practice, to avoid underdetermined fits to limited data, we set Δ*g*′′ and γ*_iv_* to zero. This model for the spectrum of surface fluctuations has small and large wave-vector regimes. At large wave vectors, the spectrum of fluctuations depends only on the stiffness and surface tension coefficients, ${\tilde{\gamma }}_{\mathrm{iw}}$ and γ*_wv_*, respectively (*36*). As in extended capillary wave Hamiltonians, these are modeled as even polynomials of *q* to order 4. Once ${\tilde{\gamma }}_{\mathrm{iw}}$ and γ*_wv_* are known, we fit the remaining parameters *g*′′ and υ to match simultaneously the low wave-vector regime of *q*^2^〈*z*_if_(*q*)*z*_if_(*q*)〉, *q*^2^〈*z*_fv_(*q*)*z*_fv_(*q*)〉, and *q*^2^〈*z*_if_(*q*)*z*_fv_(*q*)〉 as obtained from simulations. The model reproduces very accurately the stiffness coefficients at high temperature. In the region where the DOF phase appears, it does, however, not grasp the enhanced height fluctuations. However, quite generally, the correlation functions may be expressed as a spectral series. The mean field solution corresponds to the “trivial” eigenmode, which results for the quadratic expansion of Hamiltonian. Higher-order solutions yield additional eigenmodes. For strictly periodical height profiles, the spectrum of eigenmodes has a band structure. For the quasi-long range observed in the DOF phase, we expect the spectrum will not have continuous bands, but rather a region of close eigenvalues that are separated from the mean field mode. Accordingly, the effect of the inhomogeneity appears as an additive correction to the mean field solution. This allows us to obtain meaningful fits to the regular part of the spectrum merely by neglecting the strong enhancement at intermediate length scales. The step free energy for the uncoupled sine-Gordon model can be obtained from the parameters ${\tilde{\gamma }}_{\mathrm{iw}}$ and υ as $\beta_{\mathrm{iw}}=(8/q_{z}^{2}){\left(\gamma_{\mathrm{iw}}\upsilon \right)}^{1/2}$ (*36*, *41*). Because we find that the premelting film thickness is unimodal, a step on the i/f surface will provoke a similar step on the f/v surface, with a step energy of $\beta_{\mathrm{wv}}=(8/q_{z}^{2}){\left(\gamma_{\mathrm{wv}}g^{\prime \prime }\right)}^{1/2}$. The cost of creating a step on the premelting film is therefore the sum β = β*_iw_* + β*_wv_*, which we use as an estimate for the step free energy of the ice/vapor interface in Fig. 6.

## Supplementary Material

aay9322_Movie_S1.mp4

aay9322_Movie_S6.mp4

aay9322_Movie_S4.mp4

aay9322_Movie_S5.mp4

aay9322_Movie_S2.mp4

aay9322_SM.pdf

aay9322_Movie_S3.mp4

## SUPPLEMENTARY MATERIALS

Supplementary material for this article is available at http://advances.sciencemag.org/cgi/content/full/6/21/eaay9322/DC1

## References

[1] U. Nakaya, *Snow Crystals: Natural and Artificial* (Harvard Univ. Press, 1954).

[2] Kiselev A., Bachmann F., Pedevilla P., Cox S. J., Michaelides A., Gerthsen D., Leisner T., Active sites in heterogeneous ice nucleation—The example of k-rich feldspars. Science 355, 367–371 (2017). 27940582 10.1126/science.aai8034

[3] Qiu Y., Odendahl N., Hudait A., Mason R., Bertram A. K., Paesani F., DeMott P. J., Molinero V., Ice nucleation efficiency of hydroxylated organic surfaces is controlled by their structural fluctuations and mismatch to ice. J. Am. Chem. Soc. 139, 3052–3064 (2017). 28135412 10.1021/jacs.6b12210

[4] Peter T., Marcolli C., Spichtinger P., Corti T., Baker M. B., Koop T., When dry air is too humid. Science 314, 1399–1402 (2006). 17138887 10.1126/science.1135199

[5] Sei T., Gonda T., The growth mechanism and habit change of ice crystals growing from the vapor phase. J. Cryst. Growth 94, 697–707 (1989).

[6] Demange G., Zapolsy H., Patte R., Brunel M., A phase field model for snow crystal growth in three dimensions. npj Comput. Mater. 3, 15 (2017).

[7] Furukawa Y., Wettlaufer J., Snow and ice crystals. Phys. Today 60, 70–71 (2007).

[8] Bailey M. P., Hallett J., A comprehensive habit diagram for atmospheric ice crystals: Confirmation from the laboratory, AIRS II, and other field studies. J. Atmos. Sci. 66, 2888–2899 (2009).

[9] Kuroda T., Lacmann R., Growth kinetics of ice from the vapour phase and its growth forms. J. Cryst. Growth 56, 189–205 (1982).

[10] Wei X., Miranda P. B., Shen Y. R., Surface vibrational spectroscopic study of surface melting of ice. Phys. Rev. Lett. 86, 1554–1557 (2001). 11290191 10.1103/PhysRevLett.86.1554

[11] Bluhm H., Ogletree D. F., Fadley C. S., Hussain Z., Salmeron M., The premelting of ice studied with photoelectron spectroscopy. J. Phys. Condens. Matter 14, L227–L233 (2002).

[12] Smit W. J., Bakker H. J., The surface of ice is like supercooled liquid water. Angew. Chem. Int. Ed. Engl. 56, 15540–15544 (2017). 28941041 10.1002/anie.201707530

[13] Sánchez M. A., Kling T., Ishiyama T., van Zadel M.-J., Bisson P. J., Mezger M., Jochum M. N., Cyran J. D., Smit W. J., Bakker H. J., Shultz M. J., Morita A., Donadio D., Nagata Y., Bonn M., Backus E. H. G., Experimental and theoretical evidence for bilayer-by-bilayer surface melting of crystalline ice. Proc. Natl. Acad. Sci. U.S.A. 114, 227–232 (2017). 27956637 10.1073/pnas.1612893114 PMC5240679

[14] Slater B., Michaelides A., Surface premelting of water on ice. Nat. Rev. Chem 3, 172–188 (2019).

[15] Bartels-Rausch T., Ten things we need to know about ice and snow. Nature 494, 27–29 (2013). 23389527 10.1038/494027a

[16] Ball P., Close to the edge. Nature Mat. 15, 1060 (2016). 10.1038/nmat4763 27658452

[17] Libbrecht K. G., Physical dynamics of ice crystal growth. Annu. Rev. Mater. Res. 47, 271–295 (2017).

[18] Conde M. M., Vega C., Patrykiejew A., The thickness of a liquid layer on the free surface of ice as obtained from computer simulation. J. Chem. Phys. 129, 014702 (2008). 18624491 10.1063/1.2940195

[19] Qiu Y., Molinero V., Why is it so difficult to identify the onset of ice premelting? J. Phys. Chem. Lett. 9, 5179–5182 (2018). 30149705 10.1021/acs.jpclett.8b02244

[20] J. D. Weeks, The roughening transition, in *Ordering in Strongly Fluctuating Condensed Matter Systems*, T. Riste, Ed. (Plenum, 1980), pp. 293–317.

[21] Rommelse K., den Nijs M., Preroughening transitions in surfaces. Phys. Rev. Lett. 59, 2578–2581 (1987). 10035588 10.1103/PhysRevLett.59.2578

[22] Prestipino S., Santoro G., Tosatti E., Preroughening, diffusion, and growth of a fcc(111) surface. Phys. Rev. Lett. 75, 4468–4471 (1995). 10059916 10.1103/PhysRevLett.75.4468

[23] Jagla E. A., Prestipino S., Tosatti E., Surface-melting-induced preroughening. Phys. Rev. Lett. 83, 2753–2756 (1999).

[24] Woodraska D. L., Jaszczak J. A., Roughening and preroughening of diamond-cubic {111} surfaces. Phys. Rev. Lett. 78, 258–261 (1997).

[25] Weichman P. B., Prasad A., Zippering and intermeshing: Novel phase diagrams for interfaces and films. Phys. Rev. Lett. 76, 2322–2325 (1996). 10060668 10.1103/PhysRevLett.76.2322

[26] Abascal J. L. F., Sanz E., Fernandez R. G., Vega C., A potential model for the study of ices and amorphous water: Tip4p/ice. J. Chem. Phys. 122, 234511 (2005). 16008466 10.1063/1.1931662

[27] Benet J., Llombart P., Sanz E., MacDowell L. G., Premelting-induced smoothening of the ice-vapor interface. Phys. Rev. Lett. 117, 096101 (2016). 27610864 10.1103/PhysRevLett.117.096101

[28] Fletcher N. H., Reconstruction of ice crystals at low temperatures. Philos. Mag. B 66, 109–115 (1992).

[29] Buch V., Groenzin H., Li I., Schultz M. J., Tosatti E., Proton order in the ice crystal surface. Proc. Natl. Acad. Sci. U.S.A. 105, 5969–5974 (2008). 18408162 10.1073/pnas.0710129105 PMC2329717

[30] Pan D., Liu L.-M., Tribello G. A., Slater B., Michaelides A., Wang E., Surface energy and surface proton order of the ice ih basal and prism surfaces. J. Phys. Condens. Matter 22, 074209 (2010). 21386387 10.1088/0953-8984/22/7/074209

[31] Kling T., Kling F., Donadio D., Structure and dynamics of the quasi-liquid layer at the surface of ice from molecular simulations. J. Phys. Chem. C 122, 24780–24787 (2018).

[32] Dosch H., Lied A., Bilgram J. H., Glancing angle x-ray scattering studies of the premelting of ice surfaces. Surf. Sci. 327, 145–164 (1995).

[33] Espinosa J. R., Vega C., Sanz E., Ice-water interfacial free energy for the tip4p, tip4p/2005, tip4p/ice, and mw models as obtained from the mold integration technique. J. Phys. Chem. C 120, 8068–8075 (2016).

[34] H. R. Pruppacher, J. D. Klett, *Microphysics of Clouds and Precipitation* (Springer, Heidelberg, 2010).

[35] Davidchack R. L., Morris J. R., Laird B. B., The anisotropic hard-sphere crystal-melt interfacial free energy from fluctuations. J. Chem. Phys. 125, 094710 (2006). 16965108 10.1063/1.2338303

[36] Benet J., Llombart P., Sanz E., MacDowell L. G., Structure and fluctuations of the premelted liquid film of ice at the triple point. Mol. Phys. 117, 2846–2864 (2019).

[37] Buch V., Sandler P., Sadlej J., Simulations of H_2_O solid, liquid and clusters, with an emphasis on ferroelectric ordering transition in hexagonal ice. J. Phys. Chem. B 102, 8641–8653 (1998).

[38] Bussi G., Donadio D., Parrinello M., Canonical sampling through velocity rescaling. J. Chem. Phys. 126, 014101 (2007). 17212484 10.1063/1.2408420

[39] Lechner W., Dellago C., Accurate determination of crystal structures based on averaged local bond order parameters. J. Chem. Phys. 129, 114707 (2008). 19044980 10.1063/1.2977970

[40] Llombart P., Bergua R. M., Noya E. G., MacDowell L. G., Structure and water attachment rates of ice in the atmosphere: Role of nitrogen. Phys. Chem. Chem. Phys. 21, 19594–19611 (2019). 31464318 10.1039/c9cp03728d

[41] Noziéres P., Gallet F., The roughening transition of crystal surfaces. I. static and dynamic renormalization theory, crystal shape and facet grwoth. J. Phys. 48, 353–367 (1987).

