# Supplementary material for: Surface phase transitions and crystal habits of ice in the atmosphere
Source: Sci Adv. 2020 May 20;6(21):eaay9322. doi: 10.1126/sciadv.aay9322 (PMC7314560; doi:10.1126/sciadv.aay9322)
Supplement: aay9322_SM.pdf [file aay9322_SM.pdf]

Science Advances
AAAS

advances.sciencemag.org/cgi/content/full/6/21/eaay9322/DC1

Supplementary Materials for

Surface phase transitions and crystal habits of ice in the atmosphere

Pablo Llombart, Eva G. Noya, Luis G. MacDowell*

*Corresponding author. Email: lgmac@quim.ucm.es

Published 20 May 2020, *Sci. Adv.* **6**, eaay9322 (2020)
DOI: 10.1126/sciadv.aay9322

The PDF file includes:

Legends for movies S1 to S6
Figs. S1 to S7

Other Supplementary Material for this manuscript includes the following:

(available at advances.sciencemag.org/cgi/content/full/6/21/eaay9322/DC1)

Movies S1 to S6

Other Supplementary Material for this work is:

Movie S1. Local height fluctuations $\delta z_{\mathrm{if}}(\mathbf{r})$ of the i/f surface for the basal plane (see also Fig.S6).

Movie S2. Local height fluctuations $\delta z_{\mathrm{fv}}(\mathbf{r})$ of the f/v surface for the basal plane (see also Fig.S6).

Movie S3. Local fluctuations of film thickness $h(\mathbf{r})$ for the basal plane (see also Fig.S6).

Movie S4. Local height fluctuations $\delta z_{\mathrm{if}}(\mathbf{r})$ of the i/f surface for the prism plane (see also Fig.S7).

Movie S5. Local height fluctuations $\delta z_{\mathrm{fv}}(\mathbf{r})$ of the f/v surface for the prism plane (see also Fig.S7).

Movie S6. Local fluctuations of film thickness $h(\mathbf{r})$ for the prism plane (see also Fig.S7).

Supplementary Figures S1 to S7

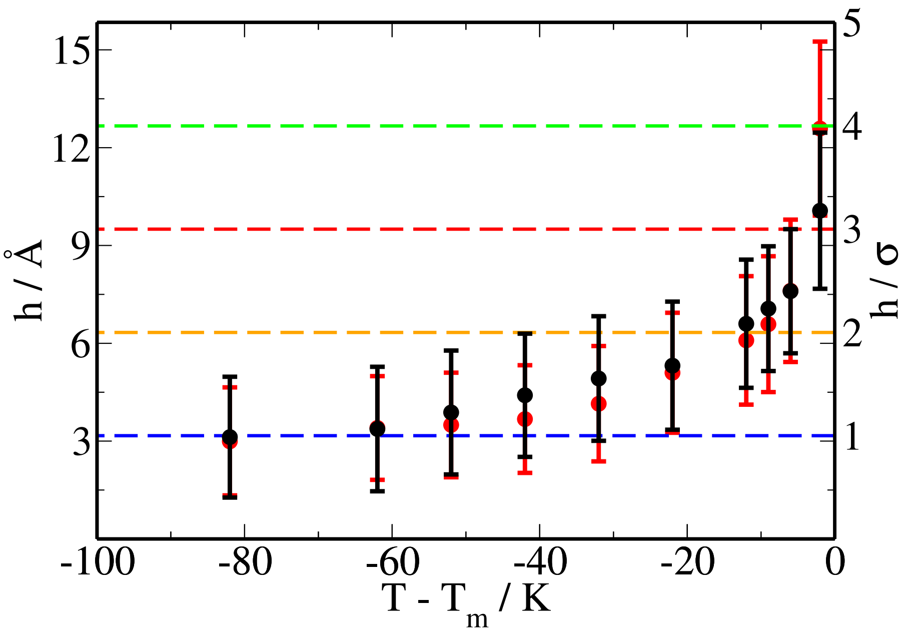

Fig.S1: Premelting film thickness as a function of temperature for basal and prism faces. Figure shows the thermally averaged film thickness h of the basal (black) and prism (red) planes. Dashed lines indicate multiples of the molecular diameter as measured in units of the Lennard-Jones σ parameter. The thickness of the prism plane remains only slightly below that of the basal plane up to 270 K, where the prism plane premelts by almost one full bilayer more

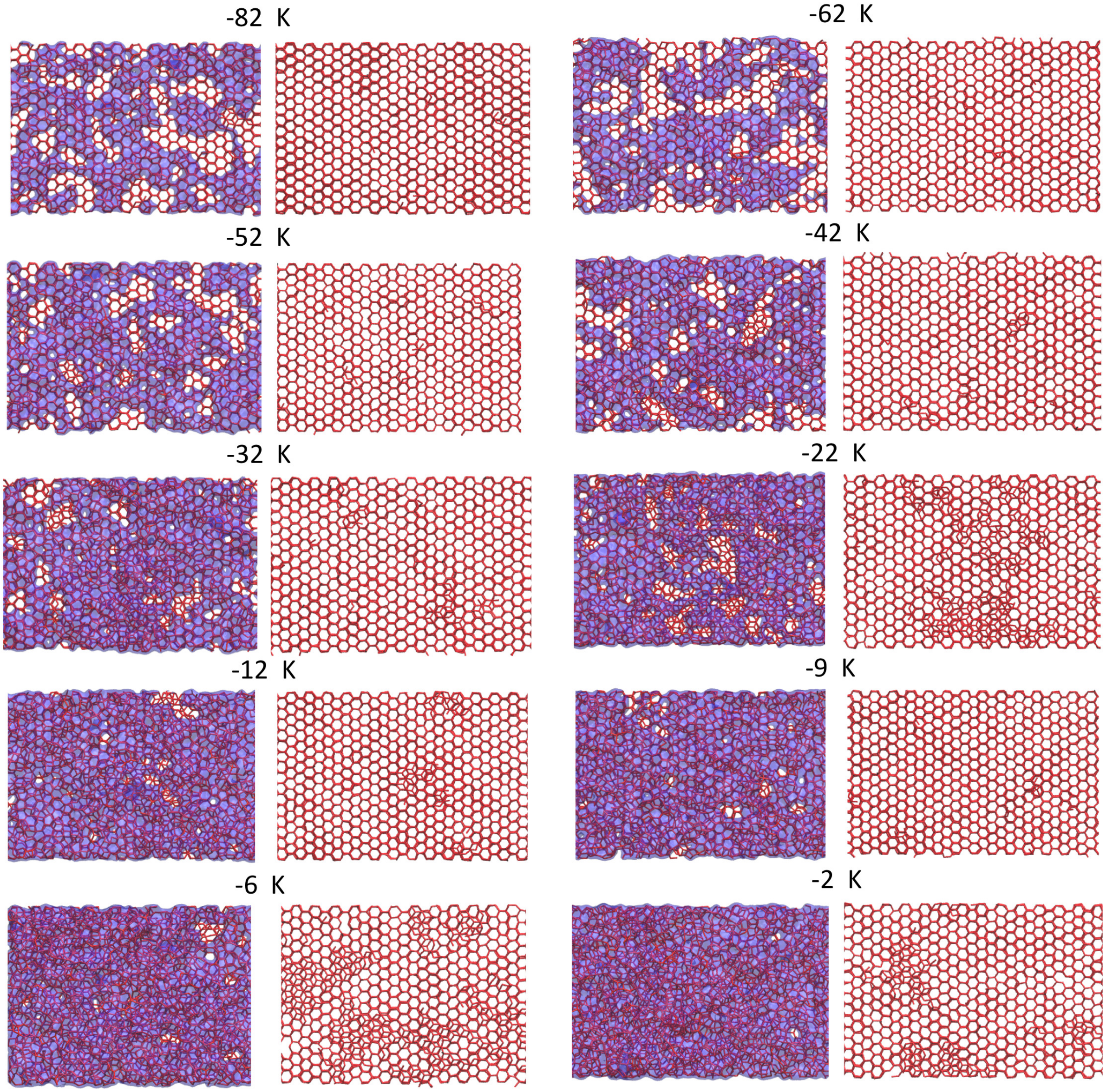

Fig.S2: Evolution of surface structure with temperature on the basal face. The first and third columns show the position of all atoms in the cluster of condensed molecules (solid-like or liquid-like), projected onto the x-y plane. The red wire-frame joins atoms separated by less than 3.5 Å, which is the same criteria used to define first neighbours in the algorithm to search for solid clusters. Liquid-like atoms are further coloured in violet. The typical hexagonal honeycomb is clearly visible on patches not covered by premelted water-like molecules. Also notice how the premelted molecules often occupy interstitial positions on the center of the hexagonal honeycomb. The second and third columns show only the positions of solid-like atoms, with liquid-like atoms left apart. As far as the position of oxygen atoms, the surface remains unreconstructed in all the temperature domain. Patches of stacking disordered ice appear at temperatures $\Delta T = -22$ and -2 K consistent with the melting of complete solid bilayers.

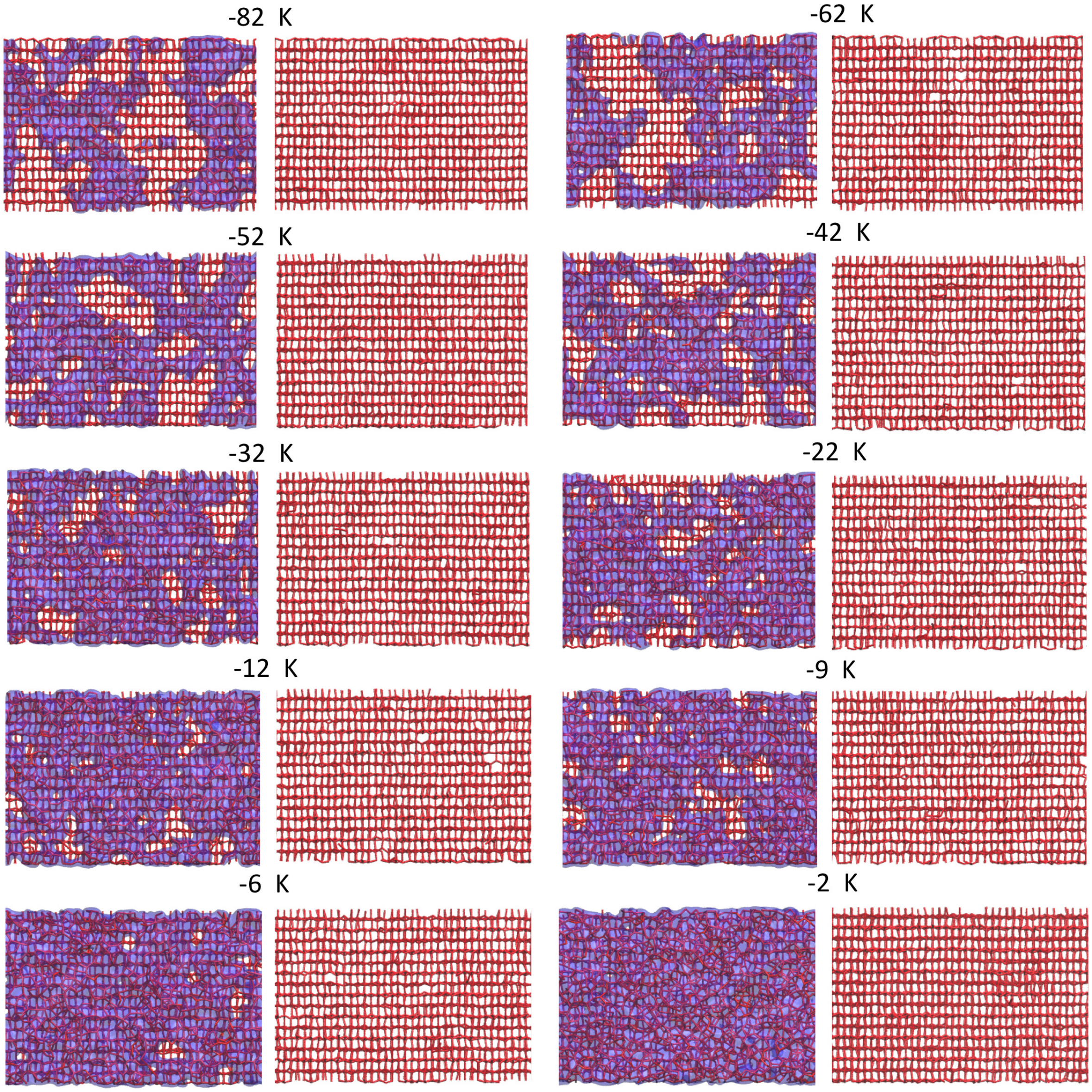

Fig.S3: Evolution of surface structure with temperature on the prism face. The first and third columns show the position of all atoms in the cluster of condensed molecules (solid-like or liquid-like) projected onto the x-y plane. The red wire-frame joins atoms separated by less than 3.5 Å, which is the same criteria used to define first neighbours in the algorithm to search for solid clusters. Liquid-like atoms are further coloured in violet. The typical rectangular structure of prism faces is clearly visible on patches not covered by premelted water-like molecules. The second and third columns show only the positions of solid-like atoms, with liquid-like atoms left apart. The oxygen framework remains unreconstructed in all the temperature domain.

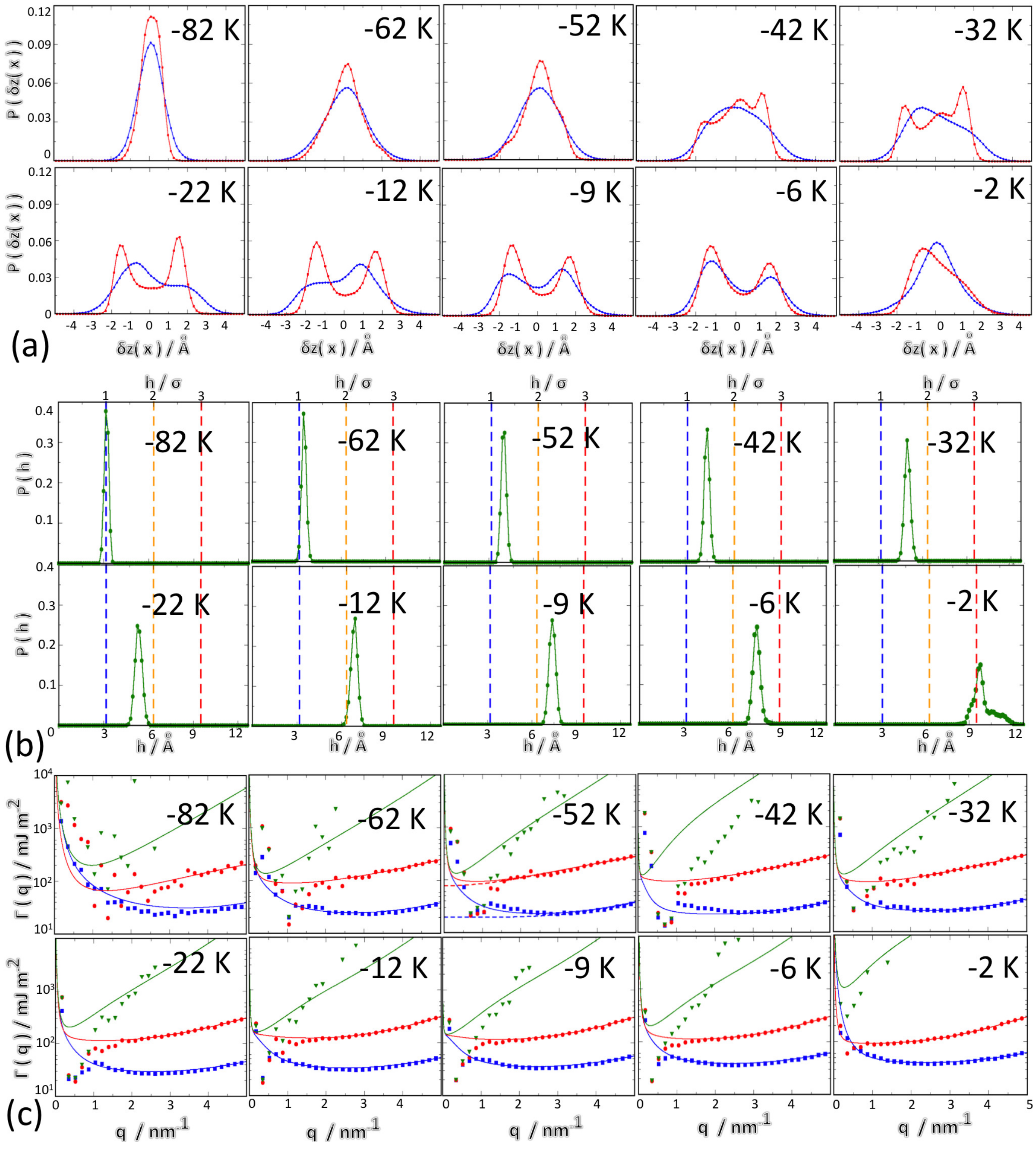

Fig.S4: Surface fluctuations on the basal face at all studied temperatures. **(a)** Probability distribution of i/f (blue) and f/v (red) surface fluctuations, as measured by the deviations of the interface position along x about the average surface. **(b)** Probability distribution of the global premelting layer thickness, h, on the basal face for several temperatures as indicated in the color code. The vertical dashed lines indicate the location of multiples of the layer thickness in units of the molecular diameter (σ parameter of the Lennard-Jones bead in the TIP4P/Ice model). **(c)** Spectrum of fluctuations on the basal face. The figure shows wave-vector dependent stiffness coefficients, as obtained from the inverse surface structure factor for i/f correlations (blue), f/v correlations (red) and crossed i/f - f/v correlations (green). Symbols are results from simulations. The full lines are a fit of the SG+CW model to the small wave-vector results, using the large wave-vector fit shown in dashed lines as input.

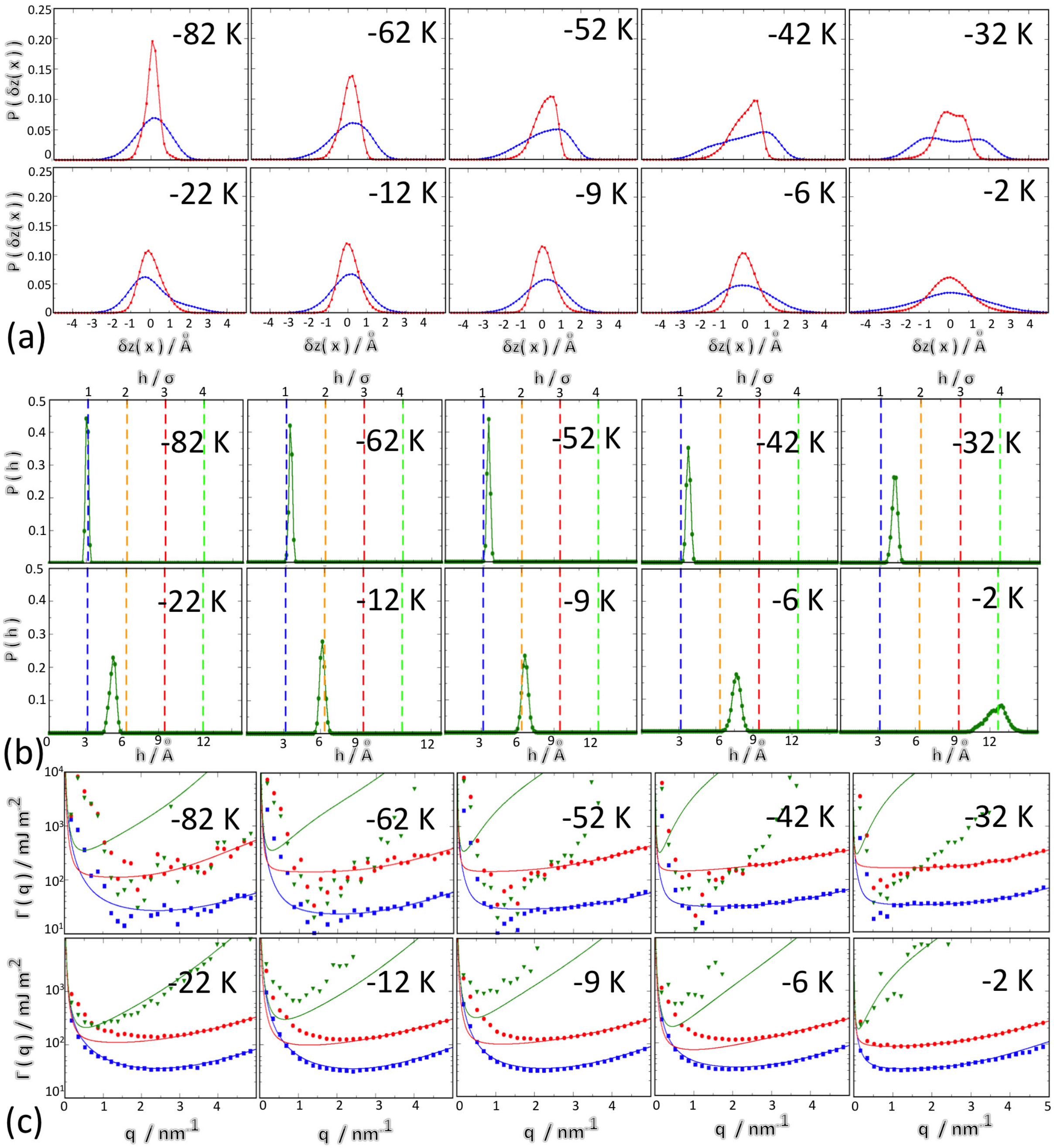

Fig.S5: Surface fluctuations on the prism face at all studied temperatures. **(a)** Probability distribution of i/f (blue) and f/v (red) surface fluctuations, as measured by the deviations of the interface position along x about the average surface. **(b)** Probability distribution of the global premelting layer thickness, h, on the basal face for several temperatures as indicated in the color code. The vertical dashed lines indicate the location of multiples of the layer thickness in units of the molecular diameter (σ parameter of the Lennard-Jones bead in the TIP4P/Ice model). **(c)** Spectrum of fluctuations on the basal face. The figure shows wave-vector dependent stiffness coefficients, as obtained from the inverse surface structure factor for i/f correlations (blue), f/v correlations (red) and crossed i/f - f/v correlations (green). Symbols are results from simulations. The full lines are a fit of the SG+CW model to the small wave-vector results, using the large wave-vector fit shown in dashed lines as input.

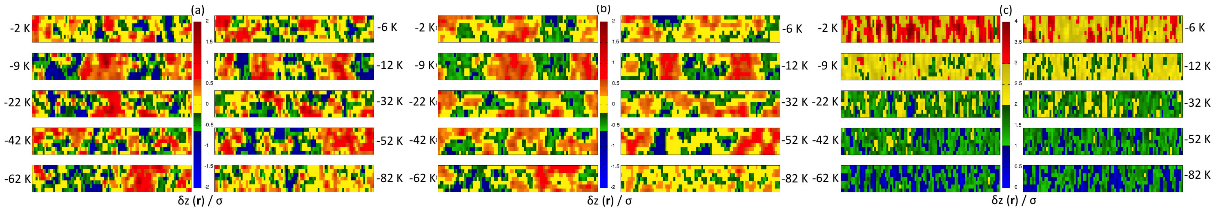

Fig.S6: Surface plots of local height fluctuations for the basal face. **(a)** Local height fluctuations $\delta z_{\rm if}(\mathbf{r})$ of the i/f surface (see Movie S1). **(b)** Local height fluctuations $\delta z_{\rm fv}(\mathbf{r})$ of the f/v surface (see Movie S2). At low and high temperatures, the correlation lengths are small and one finds alternated red and blue patches of small size. At intermediate temperatures there appear large red and blue patches indicative of the emergence of large correlations of preferred wave-length. In this regime, comparison of a) and b) shows that the i/f and f/v surfaces are also highly correlated. **(c)** Local fluctuations of film thickness $h(\mathbf{r})$ (see Movie S3). The correlation length of $\delta h(\mathbf{r})$ remains small at all temperatures as is visible from the small size of alternating patches. The thickening of the films as temperature increases is apparent from the change in color code.

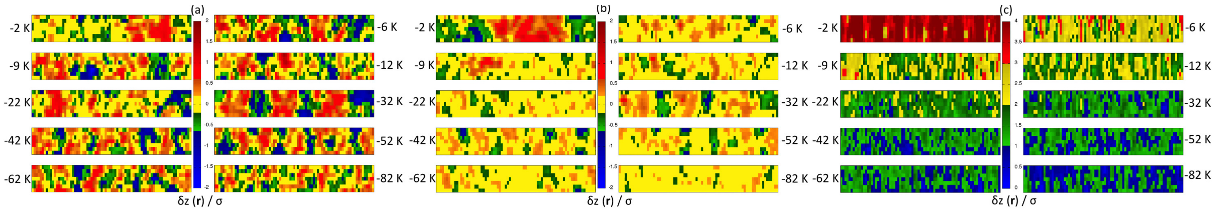

Fig.S7: Surface plots of local height fluctuations for the prism face. **(a)** Local height fluctuations $\delta z_{\rm if}(\mathbf{r})$ of the i/f surface (see Movie S4). At low temperatures the alternation of small blue and red domains is indicative of small correlation lengths. The size of the domains increases significantly at a DOF phase at T=240 K and then decreases again. At the highest temperature, T=270 K, a large correlated domain that spans most of the simulation cell is indicative of the approach to a roughening transition. **(b)** Local height fluctuations $\delta z_{\rm fv}(\mathbf{r})$ of the f/v surface (see Movie S5). The temperature evolution of correlation lengths is similar to that of the i/f , but compared to the basal surface, the amplitude of the fluctuations here is much smaller. **(c)** Local fluctuations of film thickness $h(\mathbf{r})$ (see Movie S6). As for the basal face, the correlation length of $\delta h(\mathbf{r})$ remains small at all temperatures as is visible from the small size of alternating patches. The thickening of the films as temperature increases is apparent from the change in color code.
